# Black Lives Matter and Mães de Maio: What Unites Us

**DOI:** 10.3389/fsoc.2022.699616

**Published:** 2022-05-09

**Authors:** Anelise Gregis Estivalet

**Affiliations:** ^1^University of Brasília, Brasília, Brazil; ^2^Center for Education Research at Stanford (CERAS), Stanford University, Stanford, CA, United States

**Keywords:** feminist movements, violence, Black Lives Matter, Mães de Maio, justice

## Abstract

The long history of slavery in the USA and Brazil is still evident when looking at the violence which takes place in each country today. In addition, the growing militarization of public management is due to the foreign policy of the USA and the military dictatorship of Brazil which lasted more than 30 years. Facing situations of violence, mainly state-owned, the 1970s were marked by women's resistance and struggle against violence, authoritarianism and lack of citizenship, particularly in Latin America. These social movements represented the distancing of ideology as an engine of social mobilizations, as well as the conversion of collective identity policies into generators of responses. The ability to form a collective identity around the common identification of oppression allowed the development of these new mass movements. From the construction of a collective female identity, intimate and personal aspects gained a central dimension in the identification of oppression, consequently, in the project of personal and social transformation. The agendas of this second wave of the feminist movement encompassed both the struggle for civil rights and the rights of blacks, pacifist, student and decolonization movements. Considering the influence of these new feminist movements on two current social movements, namely “Black Lives Matter” (United States) and “Mães de Maio” (Brazil), I want to understand, in this article, how the guiding meanings of gender, race, sexuality, class and generation, present in the third and fourth waves of feminists, appear in practice, in these two social movements that have the same generative facts as triggers for their constitution.

“We need to understand that women, and mothers who are on the front lines, have a struggle that comes from the womb. We are here to give birth to a new society. And perhaps, it will emerge after this pandemic. Even with fascism on the rise, perhaps people can take another look at the world” Débora Maria da Silva, leader of the Mães de Maio movement.

## Introduction

The United States of America (USA) experienced more than 100 years of slavery, whilst Brazil's slavery history exceeds 300 years. In addition, the USA and Brazil are registering an increasing militarization of public management, the result of the war policy implemented worldwide by the USA and by more than 30 years of military dictatorship registered by the Brazil. Moura et al. (2010) defines Brazil as an example of a country that is experiencing a brand new type of violent and armed conflict. Despite being a country that is not officially involved in a war, in some regions it has high rates of homicides caused by firearms. In this sense, we can say that the armed violence that affects countries like the USA and Brazil is configured in a segregation continuity.

According to a study by the United Nations Office on Drugs and Crime (UNODC), published in 2019, America is the continent with the highest percentage of intentional homicides, 37.4%. In South America, Brazil has the second-highest homicide rate on the continent (30.5 homicides per 100 thousand people). A significant portion of these records is attributed to state violence. Regarding Brazil, according to the World Health Organization (2019), the country had in 2016 the highest absolute number of homicides (64,900). In total, about 1.2 million people lost their lives to intentional homicides in Brazil between 1991 and 2017. Although the United States has registered decreasing homicide rates since 2001, Brazil has registered increasing rates in recent years. Firearms are also involved “far more often” in homicides in the Americas than in other regions, according to the UNDOC report. The similarities between Brazil and the USA do not stop there. Both countries also have historical records of lynchings. A survey by the Center for the Study of Violence at the University of São Paulo (USP), which studied cases of lynching from 1980 to 2006, found that Brazil is the country with the most lynching in the world (Souza Martins, 2015). Meanwhile, the US has a similar record, having recorded more than 4,400 lynchings for racial terrorism between 1915 and 1940 (Equal Justice Initiative, 2017).

Also, in general, the Americas still have the highest rate of victims among young adult males worldwide. According to the Anuário Brasileiro de Segurança Pública (2020), the Brazilian police murdered 6,357 people in 2019, 99% of whom were men and 79% were black. Bureau of Justice Statistics^1^ data estimates that between June 2015 and May 2016, 1,200 people were killed by police in the U.S. And in both the US and Brazil, the death rate of blacks by the police is at least twice that of whites. Data from the “Black Lives Matter: Eliminating Racial Inequity in the Criminal Justice System (Ghandnoosh, 2015)” report warn that, despite the black population representing 13% of the US population, the overall homicide rate for blacks was 6.2 times higher than for whites in 2011.

The so-called “May Crimes” in Brazil represented the most emblematic episode in this context. Between May 12 and 20, 2006, in the state of São Paulo, police and paramilitary groups acted supported by a “wave of response” to “attacks by the First Command of the Capital (PCC).” In 9 days, at least 564 homicides by firearms were committed, and many of these people are still missing, considering the occultation of corpses and falsification of reports (Amadeo et al., 2019). The May Crimes mainly victimized poor young people, usually black or Afro-indigenous descendants. However, both these murders and so many others remain forgotten, without the perpetrators being properly investigated, judged, and punished.

The “Era das Chacinas,” which began in 1990 with the Massacre of Acari, unfortunately still has its traces today. In just over 30 years, we can mention many others, most of which occurred in the Rio de Janeiro-São Paulo-Northeast Brazil axis, such as Matupá (1991), Carandiru (1992), Candelária and Vigário Geral (1993), Alto da Bondade (1994), Corumbiara (1995), Eldorado dos Carajás (1996), São Gonçalo and Favela Naval (1997), Maracanã (1998), Cavalaria and Vila Prudente (1999), Jacareí (2000), Caraguatatuba (2001), Castelinho, Jardim Presidente Dutra and Urso Branco (2002), Amarelinho, Via Show and Borel (2003), Caju and Praça da Sé (2004), Baixada Fluminense (2005), Complexo do Alemão (2007), Morro da Providência (2008), Canabrava (2009), Vitória da Conquista and the April crimes in Baixada Santista (2010), Praia Grande (2011) and Cabula (2015). This is without considering the countless homicides that occurred by what we usually call “stray bullet,” homicides that affect, in most cases, young people and children and generally occur during police actions when confronting armed groups [Defensoria Pública do Estado do Rio de Janeiro (DPERJ), 2021]. Also in these cases, the perpetrators are rarely punished. Many homicides committed by police forces in the United States seem to have the same outcome (acquittal for self-defense or manslaughter), although in recent times they have been filmed and have gained notoriety in the press. These homicides also affect, to a large extent, poor, black and peripheral youths. According to Stinson (2020), only 1% of police officers who kill civilians in the United States are (effectively) accused of murder. Stinson warns that since 2005, about 140 police officers have been arrested on charges of murder or manslaughter. Of those, only 44 were convicted, in most cases, but of a minor crime. It was in this context of impunity that movements such as Black Lives Matter (BLM) and Mães de Maio emerged.

Considering the historical context and similar aspects mentioned, whether they are the violence rates and the growing militarization of public security in both the US and Brazil, I analyze in this article the responses that emerged from social movements, specifically two women's movements: Black Lives Matter and Mães de Maio. Thus, in the next section, I debate the impact of violence in America and the new feminist movements that emerged from this condition. In a third moment, I present the two social movements considered here: BLM and Mães de Maio. In the later session, I discussed the particularities of American feminism and how this fourth wave carries specificities that are reflected in the movements analyzed here. Finally, I present final reflections on the topic are presented and conclude that the fourth wave of feminism encompasses actors who identify with agendas that had hitherto been relegated to the domestic sphere and, in the cases presented here, of activists who react to state violence.

## Violence in America and the New Feminist Movements

During the 1970s and 1980s, the second wave of feminism emerged from women's resistance and struggle against violence, authoritarianism and a lack of citizenship in military regimes (Matos and Paradis, 2013). At that time, feminisms movements were built in opposition to the state. As stated by Alvarez (2000), autonomy meant independence and opposition to the state and the left. As a movement that emerged and sought to define its contours, this definition included the organization of spaces for its specific agendas and priorities.

This new feminism appeared in a more general context than what we have come to call “new social movements” (Laraña et al., 1994). Thus, it emerged in parallel to the struggles for civil rights, black rights, pacifist movements, student movements, the new left and decolonization movements. Several studies indicate that the new social movements represented the detachment of ideology as an engine of social mobilizations, as well as the conversion of collective identity policies into generators of social responses (Melucci, 1989, 1996). The ability to form a collective identity around the common identification of women's oppression allowed the development of this new mass movement.

The Women's Liberation Movement (WLM), initiated in North America, developed from the construction of a collective female identity that gave it an extraordinary importance in combining the relationship between the individual and the collective, between the public and the private. “Precisely, the defining epicenter of this new movement was the innovative reading according to which what is personal is political, the shaking of the borders between what is public and what is private” (Nash, 2006, p. 52–53). The intimate and personal aspects took on a central dimension in the identification of female oppression and, as a consequence, in the project of personal and social transformation of women. The crucial goals of the new feminism, such as personal development, self-esteem and individual identity, were decisive in achieving women's personal liberation. In this way, freedom and autonomy were equal to equality in degree of importance as demands. While the slogan “Black is Beautiful” had been a potent strategy for the cohesion of the black American rights movement in the 1960s, WLM, by citing women as a key point, has made the movement consolidated among black women.

In the following decades, women's movements that until then felt excluded from the feminist movement's agenda until then, sought to create a collective identity that recognized both the domestic oppression of women and their creative and transformative capacities. In this sense, these new of women's movements were also guided by the organization of movements of mothers, grandparents, widows, friends and family, generally from the less favored classes, without any political experience, who started to engage in the fight against impunity, favor of social justice and historical memory. Initiatives like these have emerged in several cities around the world, such as the mothers of Plaza de Mayo, the Family Association in Chile, the Liberian Peace Movement, the National Committee of Widows of Guatemala (CONAVIGUA), the Committee of the Mothers of the missing Salvadorans (CoMadres), the Sri Lankan Mothers Front, the Russian Committee of Mothers of Soldiers, Women Strike for Peace, among others. These movements were decisive in the fight against the political dictatorship when the shared resistance against the dictatorial regime created a link between women's issues with those of other social movements, of struggle for human rights and women's rights (Jacquette, 1989). The example of the Mothers of Plaza de Mayo is well-known: based on maternal values, these women resisted the power of the state and fought the dictatorship. They have become the innovative expression of citizenship and new political practices, built on maternal values, having them as a source of political criticism, negotiation and claiming human rights (Gingold and Vázquez, 1988).

The Mothers of Plaza de Mayo developed a powerful discourse of the urgency of life on politics and maternal love on ideology (Jacquette, 1989). They appropriated the social role and traditional gender discourse about motherhood, turning it into a political weapon and radically transforming its meaning in relation to the hegemonic discourse. From the conventions of the patriarchal ideology, they assumed their identities as mothers, but they defied many of the rules of conduct implied in the gender discourse. Using their maternal role and their identity as creators of life, they transformed their personal loss as mothers into a political issue. In addition, they appropriated the public space, making it legitimate areas for expressing their pains and dramas that until then were classified as illegitimate.

These mothers inverted the traditional modalities of political practice, transforming, through the intimate expression of their maternal suffering, the personal sphere into a public manifestation of resistance, as highlighted by Feijoó (1989, p. 23): “Their peaceful resistance turned into a strong challenge to the military character of the dictatorial regime; for this reason they have been considered a significant element in the construction of civil peace in Argentina.” The Mothers of Plaza de Mayo have become an important example of maternalism in women's movements and in the development of social practices. Despite this, different researchers have highlighted that, reinforcing a gender discourse supported by traditional female roles, the debate about its consequences on the recognition of female citizenship and autonomy remains open (Aguirre, 1997). In this sense, Molyneux (1997) indicates that the selflessness of women in community causes in the Latin American context is an object of debate as to its impact on the process of individual emancipation of women.

The coverage of what we can call maternalism as “rebel mothers” was one of the characteristics of women's movements in Latin America during the 1980s. The struggle against dictatorial regimes and democratic recovery were events that marked the political orientation of Latin Americans social movements. Both right and left speeches proposed a maternalist view of women's rights (Vargas, 1997). The right highlighted their role as mothers and natural guardians of the family, while the left demarcated their place as mothers who acted in opposition to the government and institutions for the preservation of family subsistence. In any case, neither one has broken with the traditional female role and its identity designation from social motherhood.

This is the milestone in which the women's popular movement developed, characterizing a social context in which the traditional gender discourse built its options and strategies for resistance. The social reality of the feminization of poverty has led many women from the peripheries to organize themselves in defense of the survival of their families. As a result, they articulated a movement based on the positioning of gender and social materialism when the arguments of maternal values marked their social support and their emergence as agents of change. In any case, this mobilization has always been interested in community needs. In the 1990s, community interests continued to define Western women's activity agenda (Nash, 2006). It is in this context that movements such as “Mães do Rio” emerged, which were characterized by groups of women, who became active due to the massacres that took place in Rio de Janeiro in the early 1990s and in 2005—mothers of Acari, Vigário Geral, Borel, Via Show, Queimados and Nova Iguaçu and Candelária. In addition, there is the Network of Communities and Movements against Violence, which emerged in 2004, and is formed mainly by relatives of victims of police violence in slums and the MOLEQUE movement—Mothers Movement for the Rights of Adolescents in the Socio-educational System, which has been operating since 2003.

The so-called third wave, which emerged in the 1990s, sought new alternatives that combined economic growth, deepening democracy and social justice for the Americas and colonized countries. However, for many rural and urban women workers, black, indigenous and lesbian, these principles were not sufficient, nor did women want to be treated as “the others” anymore. This discussion arises in virtue of the accusation that Western feminist theories would have been built under a reductionist, distorted, stable conception and contrary to the history of women and third world feminisms, as warned by Mohanty (1988).

Fraser (2009), for insisting on considering both political as well as social and economic factors, also proposes a new feminist theory of social justice that incorporates paradoxical dimensions not addressed by liberals who emphasized justice as equity and highlighted economic redistribution as the engine of promoting equality and social justice. Given this, the proposal for a democratic justice would encompass redistribution, recognition and representation. This theory, according to Fraser (2006), would configure Westfalian democratic justice.

## May Mothers

The Mães de Maio movement is made up of mothers, family and friends of what the movement calls victims of violence in the Brazilian state, especially at the hands of the police. The idea of the name is based on the Mothers of Plaza de Mayo movement in Argentina. It emerged from the Crimes that occurred in the state of São Paulo in May 2006. Still according to Mães de Maio, the movement started from the pain of mourning generated by the loss of children, family and friends.

The movement's mission is to fight for truth, memory and justice for all victims of violence against the poor, black and indigenous population. They seek truth and justice, in particular, for those killed and missing from the crimes of May 2006 and April 2010. The report by the NGO Global Justice and the International Human Rights Clinic (IHRC), published in 2011, indicated that the existence of strong evidence of summary execution, both in the deaths registered as “murder of unknown author” and in the homicides carried out by police officers registered as “resistance followed by death.” In this sense, one of the main flags of Mães de Maio is the unarchiving and federalization of investigations, due judgment and punishment of those responsible for the crimes that occurred in May 2006 and in April 2010 in the state of São Paulo, whose investigations, for the most part, were shelved. In addition, they ask for the definitive abolition of the records of “Resistance followed by death” (São Paulo), “Auto de Resistência^2^” (Rio de Janeiro), “Resistance to Prison” (Minas Gerais) and any related expression used in the Police Reports that restrict the state to responsibility for killings.

The movement's main axes of action are: the welcoming and solidarity between family members and friends of victims of the State; systematic reporting of cases and the status of investigations and prosecutions; participation in debates, seminars, meetings, conferences; and, the organization of fighting activities, such as protests, marches, vigils and manifestos. We have always been an uprising that inspires other mothers to scream. Today, we join mothers who have had their children missing, mothers from all over Brazil, from Rio de Janeiro. Outside the country, we have a connection with American mothers, mothers of FARC victims, mothers of Cali. And in each of these women who lose their child to the State, there is a May mother” said Débora Maria da Silva, leader of the movement, in an interview with the Universa portal, in May 2020. In November 2020, city councilor Eduardo Suplicy (Partido dos Trabalhadores), filed a municipal law project to protect survivors and family members of victims of violence produced by State agents, especially by security forces. If approved, the Mães de Maio Law, as it was baptized, will offer institutional support, social protection, and medical assistance to minimize the negative impacts generated by episodes of violence.

## Black Lives Matter

In 2020, in the midst of the COVID-19 pandemic and in the face of rising rates of contamination and deaths from the disease in the United States, thousands of people marched through the streets of different American cities after the assassination of George Floyd.

According to the website of the Black Lives Matter Global Network Foundation (2021), in 2013, three black women—Alicia Garza, Patrisse Cullors and Opal Tometi—from a political will, created a project to build the movement called #BlackLivesMatter (BLM). The BLM emerged as a response to the acquittal George Zimmerman, accused by the murderer of Trayvon Martin^3^.

As the BLM gained space, the platform started to be used as an organizing tool by other groups, organizations and individuals who identified with the anti-racist agenda. The space that the BLM started to occupy helped to boost the dialogue on violence driven by the state. Particularly also the blatant ways in which black women are raped.

In August 2014, Mike Brown^4^ was murdered by police officer Darren Wilson in Ferguson. Darnell Moore and Patrisse Cullors then organized a national race during that year's Labor Day weekend. This movement was called the Black Life Matters Ride. In 15 days, the movement then developed a plan to support the demonstrations that were organized in St. Louis and which brought together more than 600 people. In addition to St. Louis, there were protests in 18 other cities. Since Ferguson, the BLM organization has realized that there was a huge pent-up demand that sought to end state-sanctioned violence against blacks. Other cases that also had great repercussions were the murders of Eric Garner ^5^ and Breonna Taylor ^6^.

According to the BLM, the movement realized the existence of significant gaps in the spaces occupied by the black movement and its leaders. For the leaders of the movement, the black liberation movements in the USA had as main leaders cisgender and heterosexual men, leaving women, queer people, transgenders among others, out of the movement or in the background. Currently, the BLM sees itself as a decentralized network that recognizes the need to centralize the leadership of queer and trans women and people, making a commitment to place those on the margins closer to the center.

## American Feminism and the Fourth Wave

The performance of American feminism is beyond social movements in the classic sense of expression. It is inserted in the discursive field of action. Currently, it is constituted as a broad, heterogeneous, polycentric, multifaceted and polyphonic field. The majority of Non-Governmental Organizations (NGOs) that emerged at the beginning of the second wave of feminism centered their activities on popular education, empowerment and awareness of women from popular classes. Some still maintain this focus, while others focus their work on promoting and monitoring gender-related legislation. Other organizations also propose to articulate grassroots work with “macro” actions, centered on public policies and other types of political-cultural intervention. Matos and Paradis (2013, p. 97) enlightens us about this:

Feminist NGOs, increasingly specialized, made progress in the introduction of gender-related issues in different programs, while in their part they relativized their role of criticism, pressure and transformation of the State. NGOs began to play an important role in strengthening social policies, while the State experienced a hollowing out of its social function (our translation).

The “devenir mujer de la politica” reflects the lack of access to politics, self-representation and representation. Minority becoming is the other name of the multitude^7^. Becoming a woman means “being a woman” based on her own historical and also local condition. Becoming a woman also means analyzing the experience from a negative and positive point of view, what we have suffered; however, also what we are able to produce and propose to all those who want to become a minority, to all others, including men. Resistance creates subjectivity, new forms of life, a new being or a new resistance, new ways of living, speaking, exchanging and loving, producing value; it is something very material and immanent. It certainly represents a way of generating life, although there are others, such as making a community, fighting together, inventing solidarity modes and changing the relationship with the other. All of this is producing life, all of this is ontology. Judith Revel highlights:

So becoming a woman in politics is another relationship in politics in which women do not want power, they do not want the Winter Palace; that does not interest them. What they want is the word, they want space, they want the common with others, who are not women. A common capable of producing new forms of life would be an ontology of infinite difference, a multitudinous difference, an ontology of the multitude (Revel, 2008, p. 121) (our translation).

It is in this sense that American feminists intend to address multiple elements in their analysis: economics, politics, bodies, subjectivities, sexualities, among others, in order to reveal the mechanisms that support inequalities and privileges.

Feminist reflections cross several social markers of difference. I refer to social and cultural categories that place actors in certain hierarchies of power, producing the “different” in relation to the elites' life model. The ideal model produces perverse imagery about the different. Feminists have called attention to social markers such as age, nationality and place of residence to question the very idea of “different,” social categorization, the vectors of power that build and maintain these hierarchies, as well as the naturalization of discrimination and social injustice. For this, the concept of intersectionality becomes crucial, as American feminism brings up debates about the heterogeneity of feminism, especially lesbian, black, indigenous and community feminisms.

The stakes of American feminism point to the revitalization of feminist practices, considering that there are people in different situations of discrimination, marginalization and social exclusion. It means that these debates have allowed the problematization of the “woman” category itself, not to talk about “women,” but to account for their vital experiences. The renewal of the “woman” imaginary allows for inflections around social heterogeneity, considering that we are people racialized with gender, with age, with privileges and/or disadvantages due to our sexuality. This heterogeneity has allowed to generate self-critical debates about its political bets. Feminists who belong to academic collectives and/or activists are also affected by the social markers of difference. Within their groups, there are discriminatory practices that reinforce the material and symbolic violence suffered by black women, indigenous people, migrants, etc.

Specifically, Latin American women have decided to renew the imaginary of “being a woman.” This process essentially goes through the deconstruction and reconstruction of the history of our ancestors, as explained by Gargallo (2007, p. 24):

Imagining implies wanting an image of oneself, a utopian image, different from the one that roles and hierarchies assign to the person. At the same time, the desire is not a desire to appropriate something or someone outside, but a desire to know and to know oneself. In this way, renewing the imaginary of being a woman on the part of a female community supposes the will to want to review itself in history, to know if there is a possibility of defining oneself as women and to propose as a full member of the human community (our translation).

For Vargas (2008, p. 142), Latin American feminisms are heterogeneous according to their spaces of action, identities and also according to the different strategies against the State.

There is not a consensus among the feminist movement on whether or not a fourth wave exists. However, Latin feminists have advocated feminist studies and theories that focus on the countries of the global south and, in particular, Latin America and the Caribbean. The idea is for a movement that has an impact from the local to the global. The strength of the global south emerged exactly from there: from the denial of the south, there was reaction and opposition to the advances of neoliberalism. The guiding sense of this new wave would be linked to a renewal with an emphasis on intersectoral, transversal and multidisciplinary boundaries among gender, race, sexuality, class and generation, as explained by Matos (2010). These circuits of feminist diffusion are operated from different horizontal currents of feminisms (black, lesbian, academic, male, etc.) that we could call feminists idestreaming or “horizontal flow of feminism” (Alvarez, 2009).

The belief in women's genetic predisposition to peace has made both women's roles and contributions historically undervalued, whether in times of war or in times of peace. For Moura et al. (2010, p. 186), this subalternity has its roots in the social construction of the meanings of war and peace, stereotypically associated with both sexes. Women have naturally been associated with informal peace, everyday peace, and men with war and formal peace.

In this sense, the assignment of differentiated social roles according to sex, tend to subordinate women and their experiences. Thus, the patriarchal power structure determines all human action, both public and private (Reardon, 1985). This designation influences our daily practices by imposing sexually defined roles, establishing a hierarchy among them, disparaging the private space in relation to the public space, cementing and naturalizing the power relations valid in contexts of war and peace. It is these same dominant sexual representations that make possible and normalize acts of private violence, as well as acts of organized violence, appealing to myths that legitimize a romanticized and noble view of war that elects violence not only as a way of resolving disputes and guarantee of security (Tickner, 1991) as legitimizing death. Ideals such as progress, reason and civilization also justify the use of violence. Thus, peace must be kept in the background until the “natural state of affairs” is achieved. To do so, using “law and order” in the case of the USA and “order and progress” in the case of Brazil becomes a necessary action in the search for peace and its powerful racial and colonial dimension. But, what is the law in a context in which communities are disproportionately incarcerated and in which direct violence is mobilized in a discriminatory manner against certain bodies and certain people?

Moser and Clark (2001) point out that from the observation that the patriarchal system determines structural and cultural factors of violence and from the concrete analysis of the violence suffered by women, feminists established a continuum between the various types of violence and injustices that exist:

In this way, the traditional concepts of war and peace, considered as artificial and reductive, are questioned and their perversities are exposed: they neglect structural and cultural violence, which operate in the long run and are the basis of many of the expressions large-scale violence, thus naturalizing micro-violence, felt in the interpersonal sphere (not exclusively by women, but above all by them) and common globally, which constitute one of the axes of feeding new spirals of violence (Moura et al., 2010, p. 187) (our translation).

According to Schild (1998), the neoliberal state takes on a double task: on the one hand, it accentuates its punitive character, adopting security policies as part of the social agenda and, on the other hand, it takes on the face of a “caring state” with the proliferation of social policies and money transfers aimed at combating poverty, with women mothers as the main beneficiaries. This double facet of the neoliberal state is added to the processes of the women's and feminist movements and their distinct positions in a pragmatic view that proposes demands for gender equality in a clearly weakened state (precisely by neoliberalism) with postures that mark its autonomy in the face of “Patriarchal state.” For Tatiana Moura and others:

As activists for human rights and activists for justice and truth, women have also put pressure on governments, warring factions and international actors, seeking to raise awareness of the importance of historical knowledge of the scope of human rights violations and the need to establish accountability (Moura et al., 2010, p. 189) (our translation).

Thus, women's movements also propose their own agenda in favor of joining social struggles with other movements against the hegemonic neoliberal project. Alvarez (2000, p. 14) explains: “That is, the neoliberal state is also a site of cultural production, a crucial site where gender is constructed, where gender relations are re-signified, recoded and reconfigured.”

Based on this diagnosis, about the origin and dissemination of the various forms of violence, feminists ask about the effectiveness of the responses traditionally used and which are materialized in the concept of security. Starting from the idea that the conventional security paradigm constitutes a generator of insecurities, particularly at the individual level, a concept of security is proposed that transcends the traditional statocentric level so that it starts to have a multidimensional, multiscale and proportional perspective the expansion of the concept of violence (Tickner, 2001).

The struggle of women and, particularly, of mothers, tells us of the attempts to register certain deaths, which until then would have been considered unimportant, in the public space as “casualties” of war, seeking to pay attention to the sign of the social location of the bodies of the victims, as well as its moral and affective condition, as is the case with the slogan “Black lives matter.” In this war, which increasingly reaches degrees of illegitimacy, mourning, by mourning the dead publicly, not only challenges political limits (Butler, 2004) but also allows those who remain to transform it into resistance. In this sense, between different forms and dimensions of mourning—as an individual and, at the same time, social process—the action moves between personal pain and collective causes and between suffering and rights. It is a fact that both the denial of victim status to the disappeared/murdered and the difficulty of proving his authorship, makes it impossible for the victim's family and friends to comply with a grieving process. Thus, by taking their indignation to the public arena, these actors contest the justice of war that places the most vulnerable in the place of the enemy to be killed and fought. Based on this, both the argumentative work done at the various protest sites and the judicial struggle to condemn police officers is based on the importance of proving that the dead were “honest” and not “bandits” or “drug dealers”^8^:

In other words, to insert them primarily in the same rightful place as those that must be protected—and not annihilated—by the State, embodied here in the police. His challenge is woven, therefore, necessarily using a symbolic perspective marked by gender, through moral and emotional languages that perform the bankruptcy of this male who attacks instead of protecting and who brings war “home” (Vianna and Farias, 2011, p. 95) (our translation).

In denouncing the inseparability of the unequal conditions that cross bodies and territories, as well as their lack of interest in determining the conditions of deaths, these women denounce the cruel processes of producing bodies and expendable lives. In addition, as the Mothers of Plaza de Mayo, they seek to reconstruct the facts from their own reports, remaking history differently from what appears in the newspapers.

## Final Reflections

Considering the above findings, I realize that the movements mentioned here and which had women as political protagonists, are able to symbolically encompass other activists who identify with the same agendas, whether family members or not of the victims, men or women, people of different age groups or ethnic groups, who rise politically through close connections. When speaking of order, hitherto domestic, that was brutally undone by the murders that occurred, most of the time, by the hand of the State, these women bring the feminine not in their individual bodies, but as a sign of meaning in the relationships that have broken down, as well as illegitimate violence (Vianna and Farias, 2011).

Finally, since the assassination of George Floyd, at least a 100 laws have been passed in different states, pointing to changes in public safety institutions. Thus, I conclude that, mainly, after the emergence of Black Lives Matter, the USA managed to advance in the accountability of those involved and in the effectuation of agreements with pecuniary retribution for the victims' families. Unfortunately, in the case of Brazil, in addition to the country not moving in the same direction, there is greater liberalization in relation to the purchase and release of the use of firearms.

## Author Contributions

The author confirms being the sole contributor of this work and has approved it for publication.

## Conflict of Interest

The author declares that the research was conducted in the absence of any commercial or financial relationships that could be construed as a potential conflict of interest.

## Publisher's Note

All claims expressed in this article are solely those of the authors and do not necessarily represent those of their affiliated organizations, or those of the publisher, the editors and the reviewers. Any product that may be evaluated in this article, or claim that may be made by its manufacturer, is not guaranteed or endorsed by the publisher.

## Footnotes

^1^https://www.bjs.gov/ Accessed in April 2021.

^2^Auto de resistência is the official nomenclature that began to be used in 1969 by the Brazilian police to define the deaths and injuries that occurred in confrontation, resulting from the resistance to the police authority.

^3^Trayvon Martin was shot by the volunteer security guard George Zimmerman on February 26, 2012, when he was returning to his father's home in Sanford, Florida. At the time, Zimmerman chased Martin with the car, on suspicion that the boy was responsible for a series of break-ins in the region. The guard then shot Martin in the chest, who was 17, claiming self-defense. Zimmerman was questioned by the police at the time of the crime, but was released without formal charges. In 2013, the guard was cleared of charges of second-degree murder and manslaughter.

^4^Michael Brown Jr., then 18, was killed with six shots. The United States Department of Justice subsequently concluded that Officer Darren Wilson acted in self-defense.

^5^Eric Garner was killed in July 2014 after being strangled by a New York Police Department officer. Before losing consciousness, Garner spoke the phrase “I can't breathe” 11 times. NY police approached Garner on suspicion of selling “loosies” (individual cigarettes) without tax stamps. The Richmond County grand jury decided not to indict the officer responsible for the murder. Months later, New York City paid the Garner family $ 5.9 million.

^6^Breonna Taylor was 26 years old and was shot dead inside her apartment after a police raid in Louisville. The crime took place at dawn on March 13, 2020. In September of the same year, the jury decided to indict only one of the three policemen involved in the crime on three counts of first-degree “arbitrary conduct.” The other two officers who also fired were not charged. The indicted police officer was released on bail. The case led to the approval of the “Breonna Law,” which banned the invasion of homes in search operations and the admission of a black police chief in Louisville.

^7^Multitude is a biopolitical set of singularities that work and are oppressed, that resist with bodies and that, with intelligence, want to revolutionize the world. The concept of the global crowd, planted in the reality of Latin America, is firmly defined as a march toward the freedom of bodies and as violence of an unbearable need (Cocco and Negri, 2005, p. 73).

^8^In the case of Jorge Floyd, the Minneapolis police argue that his death was caused by an overdose, even though Officer Derek Chauvin was kneeling under his neck for nine minutes and twenty-nine seconds. Similarly, in the case of João Alberto Freitas, murdered by Carrefour security guards in November 2020 in southern Brazil, there were attempts to disqualify him as a victim.
